# Intramural hematoma in the proximal sealing zone of the thoracic endovascular aneurysm repair: frequency and safety in acute and subacute type B dissections

**DOI:** 10.3389/fcvm.2023.1279830

**Published:** 2023-11-20

**Authors:** Mario Lescan, Migdat Mustafi, Julia Hahn, Christian Schlensak, Mateja Andic

**Affiliations:** Department of Thoracic and Cardiovascular Surgery, University Medical Centre Tübingen, Tübingen, Germany

**Keywords:** aorta, dissection, endovascular, thoracic, stent-graft, migration, TEVAR

## Abstract

**Introduction:**

To assess the outcomes after thoracic endovascular aneurysm repair (TEVAR) in the presence of intramural hematoma (IMH) in the proximal sealing zone.

**Material and methods:**

Patient data were retrospectively extracted from the hospital records of patients treated with TEVAR for acute and chronic aortic dissection type B in one single center. The initial, preoperative, first postoperative, and last follow-up CT scans were evaluated in the aortic 3D multiplanar reformats and the centerline regarding IMH presence in the proximal sealing zone, anatomical preconditions, and the morphological TEVAR complications including migration and bird-beak. Groups with (IMH) and without IMH (no-IMH) were compared.

**Results:**

Overall, 84 patients (IMH:42; no-IMH:42) were treated at the age of 63(55; 72) years, of whom 23/84 (27%), 34/84 (40%), and 27/84 (32%) were in the hyperacute, acute and subacute dissection phases, respectively. The bovine arch was found in 10/84(12%) and the type III arch was most common (43/84;51%). IMH maximum extent was found in zones 0, 1, 2, and 3 in 14/84 (17%), 17/84 (20%), 18/84 (21%), and 6/84 (7%), respectively. Sealing was achieved in zone II in 71/84 (85%) and LSA was revascularized in 66/84 (79%) of the overall cohort. Early mortality and paraplegia were 2/84 (2%) each; stroke rate was 3/84 (4%). During the 22 months median follow-up (22;4;43) no RTAD was observed. Migration ≥10 mm (IMH: 11/82; no-IMH: 10/82; *P* = 1.0) and bird-beaks (IMH: 10/82; no-IMH: 12/82; *P* = 0.8036) were comparable in both groups and accompanied by a low aorta related mortality (1/82) in both groups.

**Conclusion:**

The presence of the IMH in the proximal TEVAR sealing zone is frequent and may not be relevant for the occurrence of the RTAD, stent-graft migration, or bird-beak formation.

## Introduction

The endovascular treatment of type B dissections (TBD) has evolved to the invasive therapy of choice in the current guidelines (1). Thus, an increasing number of patients are treated in the hyperacute and acute phases due to complicated TBD with acute life-threatening states including organ malperfusion, rupture, and conservatively unmanageable recurrent pain or uncontrollable hypertension. Furthermore, in the presence of the risk factors for aortic growth during the follow-up, TEVAR should be considered in the subacute phase to ensure aortic remodeling and prevent rupture and mortality at a later stage (2, 3).

However, TEVAR may also be associated with a higher perioperative risk, particularly when performed in the acute and hyperacute phases (4). Retrograde type A dissection (RTAD) is one of the most serious complications, associated with high mortality (4). Responsible for the occurrence of RTAD may be the vulnerability of the aortic wall in and proximal to the TEVAR sealing zone in the aortic arch in combination with excessive oversizing (4, 5). Therefore, reduced proximal oversizing has been recommended and broadly applied (6). However, reduced oversizing may create new challenges after the absorption of the IMH in the proximal sealing zone, and result in proximal stent graft malapposition, proximal sealing zone dilatation and stent-graft migration (7).

The aim of our study was to evaluate the rate of IMH in the proximal TEVAR sealing zone and to examine the impact of its presence on the occurrence of RTAD, the remodeling of the descending aorta, and the development of migration and the bird-beak configuration.

## Materials and methods

The study was approved by the local ethical committee of the University medical center Tübingen (322/2022BO2). Patient consent was waived due to the retrospective character of the study.

### TBD treatment protocol and cohort specifications

The center's protocol provides the TEVAR treatment of all complicated TBD with rupture or malperfusion in the hyperacute phase. In case of complicated TBD with uncontrolled hypertension, recurrent pain, or early aortic diameter progress of 5 mm in the CT scan 48 h after the diagnosis the invasive treatment is performed in the acute phase. Uncontrolled hypertension is defined as the inability to control blood pressure with intravenous antihypertensive therapy (targeted blood pressure <120/80 mmHg) or to substitute intravenous antihypertensive therapy with oral medication after 1-week post admission. Uncomplicated TBD patients with the presence of aortic growth risk factors including proximal entry tear diameter >10 mm, or aortic diameter >40 mm are discharged from the hospital after the oral blood pressure control has been established. Those patients are subjected to elective TEVAR in the subacute phase. All patients with acute TBD are followed up with contrast-enhanced CT scans at 48 h and 7 days on a regular base. The further follow-ups are performed at 3, 6, 12 months and yearly thereafter.

**Figure 1:**
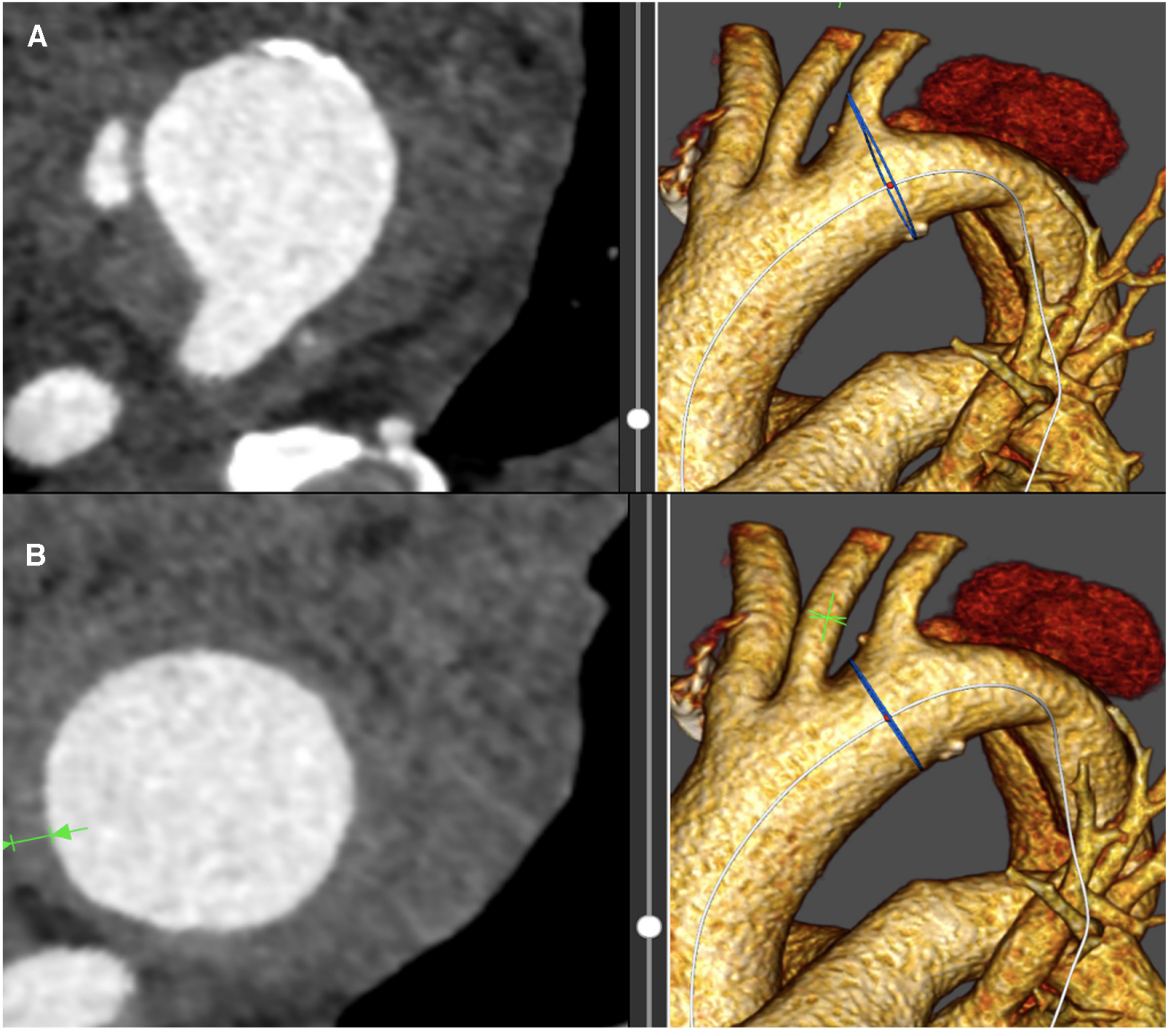
(**A**) Shows the intramural hematoma with proximal dissection extent in the aortic centerline at the site of the left subclavian artery. (**B**) demonstrates the intramural hematoma extent in the proximal landing zone II between the left carotid and left subclavian artery.

This retrospective cohort study included patients with hyperacute (<24 h), acute (day 1–14), and subacute (day 15–90) TBD treated between 2016 and 2023 with TEVAR in a tertiary referral hospital. The procedures were isolated through the search of the center's database (SAP, Walldorf, Germany). The patients were assigned to 2 study groups: The no-IMH group without the IMH in the proximal TEVAR sealing zone, and the IMH group where sealing was performed in the presence of the IMH (Figure 1). Furthermore, at least 10 mm of IMH-free sealing zone was defining the no-IMH group (8). The study design and the manuscript were organized according to the STROBE guidelines for observational studies (9).

### Population demographics, co-morbidities, and procedural specifications

Demographic data included age and sex. The co-morbidities were obtained from the hospital records: hypertension, current nicotine abuse, orally or insulin-treated diabetes, dyslipoproteinemia, COPD, previous percutaneous coronary intervention (PCI) or coronary artery bypass graft (CABG), and previous ascending aorta replacement.

Procedural details including the indication for treatment, the Ishimaru landing zone (zone 0-III), stent-graft type and diameter, the revascularization of the left subclavian artery, the presence of a bovine arch, the arch type (type I-III) (10), and the technical success were drawn from the operation protocols, intraoperative imaging, and the pre-and postoperative CT scans (11). Our procedural protocol includes the proximal oversizing in dissections of 10% and the standard use of Relay NBS (Terumo Aortic, Inchinnan, UK) and Gore C-TAG conformable (Gore Medical, Flagstaff, AZ, USA) stent-grafts without a long proximal bare stent. In the case of Relay NBS, tapered stent-grafts are used in dissections by default to reduce distal oversizing. Our preference for the Relay NBS graft can be explained by the proximal deployment mechanisms of this endograft which include the proximal to distal deployment, stabilization wires, and the V-patch in the inner aortic curvature to stabilize the endograft and to prevent the bird-beak during the deployment, respectively (12). Thanks to the V-patch the inner portion of the endograft can be securely apposed to the inner aortic curvature. In comparison to the Relay NBS, C-TAG conformable device with the active control system allows for the inner curvature apposition by the active endograft orientation feature in the proximal landing zone (13). C-TAG conformable is predominantly used in our center for patients with high true lumen tapering to address the risk of distal stent-induced new entry due to the fact that this endograft may have a reduced risk of this complication during the follow-up in comparison to the ring stent design (14).

According to our protocol, the left subclavian artery (LSA) is revascularized in all hemodynamically stable patients during the same procedure. All patients treated electively in the subacute phase receive a cerebrospinal fluid drain on the day before the operation. The endograft deployment was performed in all patients under the left ventricular output reduction with rapid pacing.

### CT analysis

CT scan analysis of the admission/preoperative, first postoperative, and last follow-up CT scan was performed with dedicated software (Therenva, Rennes, France). All patients were subjected to a contrast-enhanced CT scan with a slice thickness of 1 mm. The post-processing of the DICOM data set included the centerline measurement of the diameter of the proximal landing zone in the preoperative CT scan and the outer-to-outer total aortic diameter and TL diameters at the level of the pulmonary artery bifurcation. Proximal oversizing was calculated according to the formula:

[*(proximal stent-graft diameter, mm/outer-to-outer proximal sealing zone diameter, mm)-1] × 100%*.

Stent-graft migration was measured by the increase in the distance between the distal left common carotid artery (LCCA) origin and the proximal stent-graft end at the outer curvature of the aorta. The bird-beak was described by the angle between the innermost proximal stent-graft plane and the inner aortic curvature plane, as described in our previous works (15, 16). The CT morphological absence of the contrast agent in the venous phase defined the total false lumen thrombosis, whereas partial thrombosis included patent and thrombosed areas of the FL.

### Outcome parameters

Early follow-up outcomes included the postoperative results within 30 days after the operation, while mid-term follow-up outcomes described the findings of the last follow-up, which consisted of a contrast-enhanced CT scan and a patient interview with the physical examination. Primary technical success was reported according to the SVS reporting standards for TEVAR (11). A stroke was defined as a new neurological event that persisted for >24 h and affected the National Institutes of Health Stroke Scale (NIHSS) by at least 2 points (17). Any new onset of transient or permanent paraplegia or paraparesis after TEVAR, which occurred as a deficit in motor or sensory function of the lower extremity, or incontinence was assessed.

### Statistical analysis

The statistical analysis was performed with JMP® 14 software (SAS, NC, USA). Categorical variables are presented as patient count (percentage), and continuous variables are reported as median (1st quartile; 3rd quartile). Fisher's exact test or *x*^2 −^test was employed for categorical variables. Continuous data were tested for normality and equality of variance by Kolmogorov-Smirnov and Levene tests, respectively. *t*-test was used for normal distribution, and the Mann-Whitney U test was applied for non-normal continuous variables. Multivariate logistic regression analysis with the Wald test and likelihood ratio test was performed to assess the risk factors for migration. *P* < 0.05 was considered significant.

## Results

### Patient cohort and procedural parameters

The median age of the cohort was 63 (55; 72; Table 1) years and 21/84 were female. Between the study groups there was a trend towards a higher rate of hypertension (37/ 42 vs. 42/42; *P* = 0.0551) in the IMH group and hypercholesterinemia with statin treatment was more common in the no-IMH group (9/42 vs. 2/42; *P* = 0.0485). Other comorbidity parameters were comparable between the study groups including current nicotine abuse, diabetes, COPD, previous PCI/CABG, and previous ascending aorta replacement (Table 1).

**Table 1:** Demographic characteristics and co-morbidity of the cohort.

Patient characteristics	Overall	No-IMH	IMH	*p*
*n*	84 (100)	42 (50)	42 (50)	
Age (years)	63 (55;72)	61 (53; 71)	65 (56;73)	0.2201
Sex (female) *n* (%)	21 (25)	7 (17)	14 (33)	0.1295
Hypertension *n* (%)	79 (94)	37 (88)	42 (100)	0.0551
Current nicotin abuse *n* (%)	15 (18)	7 (17)	8 (19)	1.0
Diabetes *n* (%)	4 (5)	2 (5)	2 (5)	1.0
Hypercholesterinemia with statin treatment *n* (%)	11 (13)	9 (21)	2 (5)	0.0485
COPD *n* (%)	6 (7)	3 (7)	3 (7)	1.0
Previous PCI/CABG *n* (%)	2 (2)	2 (5)	0 (0)	0.969
Previous ascending replacement *n* (%)	2 (2)	2 (5)	0 (0)	0.969

IMH, intramural hematoma; COPD, chronic obstructive pulmonary disease; PCI, percutaneous coronary intervention; CABG, coronary artery bypass grafting.

The aortic arch types I, II, and III, were found in 20/84, 21/84, and 43/84 of the cohort, respectively, and the distribution was comparable between the study groups (*P* = 0.7219). The bovine arch was present in 10/84 patients (Table 2).

**Table 2:** Planning and procedural parameters of the cohort.

Patient characteristics	Overall	No-IMH	IMH	*p*
Arch type				
I *n* (%)	20 (24)	9 (21)	11 (26)	0.7219
II *n* (%)	21 (25)	12 (29)	9 (21)	0.7219
III *n* (%)	43 (51)	21 (50)	22 (52)	0.7219
Bovine arch *n* (%)	10 (12)	4 (10)	6 (14)	0.7379
Indication for hyperacute/acute TEVAR				
Malperfusion *n* (%)	29 (35)	21 (50)	8 (19)	0.0054
Rupture *n* (%)	18 (21)	8 (19)	10 (24)	0.7909
Recurrent pain *n* (%)	16 (9)	6 (14)	10 (24)	0.4052
Uncontrolled hypertension *n* (%)	6 (7)	2 (5)	4 (10)	0.6758
Early diameter progress ≥5 mm *n* (%)	19 (23)	6 (14)	13 (31)	0.1163
Indication for subacute TEVAR				
Aortic Diameter >40 mm at dissection onset *n* (%)	27 (32)	11 (26)	16 (38)	0.3502
Entry Diameter >10 mm at dissection onset *n* (%)	23 (27)	8 (19)	15 (36)	0.1412
Temporal dissection classification at TEVAR				
Hyperacute	23 (27)	11 (26)	12 (29)	0.1541
Acute	34 (40)	21 (50)	13 (31)	0.1541
Subacute	27 (32)	10 (24)	17 (41)	0.1541
LSA revascularisation (TEVAR with LSA coverage)	66 (79)	34 (81)	32 (76)	0.7909
Intended proximal oversizing	11 (8;13)	10 (9;13)	11 (7;13)	0.6413
Stent-graft type	43 (51)			
C TAG	8 (10)	3 (7)	5 (12)	0.7126
Relay NBS	76 (90)	39 (93)	37 (88)	0.7126
Primary entry localisation (zone)				
II	30 (36)	19 (45)	11 (26)	0.1850
III	39 (46)	17 (40)	22 (52)	0.1850
IV	15 (18)	6 (14)	9 (21)	0.1850
Proximal landing zone				
I	3 (4)	1 (2)	2 (5)	0.8405
II	71 (85)	36 (86)	35 (83)	0.8405
III	10 (12)	5 (12)	5 (12)	0.8405
Most proximal IMH extent *n* (%)				
I	17 (20)	0 (0)	17 (40)	<0.0001
II	18 (21)	8 (19)	10 (24)	<0.0001
III	6 (7)	5 (12)	1 (2)	<0.0001
0 (BCT)	9 (11)	0 (0)	9 (21)	<0.0001
0 (ascending)	5 (6)	0 (0)	5 (12)	<0.0001
IMH absent	29 (35)	29 (69)	0 (0)	<0.0001
IMH dynamics in subacute dissections *n* (%)				
Extent increase	1 (4)	0 (0)	1 (6)	1.0
Extent decrease	0 (0)	0 (0)	0 (0)	1.0
Extent stability	22 (96)	7 (100)	15 (94)	1.0
Technical success	84 (100)	42 (100)	42 (100)	1.0

IMH, intramural hematoma; TEVAR, thoracic endovascular aneurysm repair; LSA, left subclavian artery; BCT, brachiocephalic trunk.

The individuals were treated in the hyperacute, acute, and subacute dissection phases in 23/84, 34/84, and 27/84 of the cases, respectively. The most important TEVAR treatment indications in the hyperacute and acute phases were malperfusion (29/84), early diameter progress (19/84), rupture (18/84), recurrent pain (16/84), and uncontrolled hypertension (6/84). In the subacute phase, patients were treated due to the aortic diameter over 40 mm at dissection onset (27/27), and additionally, the proximal entry tear diameter over 10 mm was found in 23/27 cases.

Primary entry tear localization was zone II, III, and IV in 30/84, 39/84, and 15/84, respectively. The proximal sealing zone was zone I in 3/84, zone II in 71/84, and zone III in 10/84 of the patients. Patients acutely treated in the zone I received a carotid-carotid bypass through the ante-tracheal approach (1/84), whereas for the elective treatment in the subacute phase, a proximal scallop TEVAR for the LCCA was customized (2/84). LSA revascularization with a carotid axillary bypass was performed in 66/84 patients prior to the TEVAR but during the same intervention. The proximal oversizing of the stent-graft was 11% (8; 13), and the most common stent-graft in this study was Relay NBS (Terumo Aortic, Inchinnan, UK), which was used in 76/84 of the cohort. In 8/84 Gore CTAG conformable stent-graft was implanted. The technical success of the procedure was achieved in 84/84 (100%).

### IMH extent and dynamics

**Figure 2:**
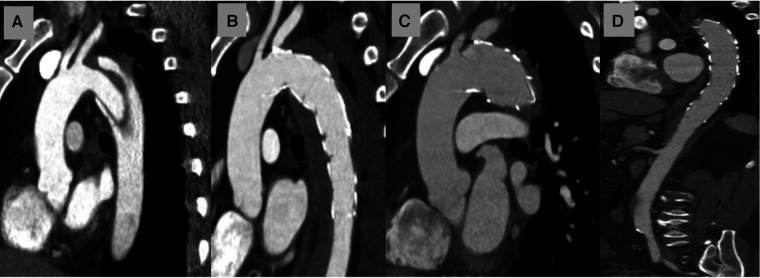
(**A**) Shows the preoperative sagittal CT scan at the time of the type B dissection diagnosis in a patient with intramural hematoma (at the site of the left subclavian artery). (**B**) demonstrates the postoperative sagittal CT scan after TEVAR in the landing zone II. (**C**) indicates the sagittal CT scan of the same patient: the stability of the proximal endograft position is well visible. The remodeling of the downstream aorta is shown in (**D**).

The presence of the IMH in the aortic arch zones 0-III affected 55/84 individuals (Figure 2). The most proximal IMH extent is shown in Table 2. In 14/84 patients the IMH reached zone 0, of whom 5/17 had a hematoma of the ascending aorta. Zone I was affected in 31/84 cases, whereas an IMH was present in zone II in 49/84. Naturally, the extent of the IMH was more proximal in the patients from the IMH group (*P* < 0.0001) but was also found in zones II (8/42) and III (5/42) of the no-IMH group. Those patients were assigned to the no-IMH group due to an IMH-free proximal sealing zone length ≥10 mm.

The patients who were treated electively in the subacute phase (median 31 days; 21; 138) had an IMH in 23/27 cases at the diagnosis CT scan. The IMH remained stable until the treatment in 22 (96%) patients and showed an extent increase in only one case.

### Early follow-up outcomes

At 30 days, mortality occurred in 2/84 (Table 3) patients. One patient with ruptured acute TBD died on the day of the operation, due to the continuous distal perfusion of the false lumen rupture site in the descending aorta. The second patient developed an infrarenal abdominal rupture of the false lumen on the second postoperative day. Stroke was found in 3/84 (4%), of whom all were non-disabling. One patient with vertebral artery transposition had a minor cerebellar stroke with transient vertigo. A second patient with a covered left vertebral artery arising from the aortic arch experienced postoperative delirium, which gradually disappeared, and a third patient, without LSA revascularization prior to the TEVAR, had a smaller posterior stroke, and recovered completely during the hospital stay. Furthermore, 2/84 (2%) patients showed postoperative paraplegia. In both cases, cerebrospinal fluid drainage was not implemented before TEVAR, due to the aortic rupture. Both had an aortic coverage over 25 cm and the LSA was covered without revascularization. One patient recovered during the hospital stay after the implementation of the cerebrospinal fluid drainage, whereas the other remained permanently paraplegic. During the early follow-up RTAD, and type I endoleaks were not observed. The median ICU stay was 0 (0;1) days and the incidence of bird-beaks was low (2/84; 2%; Table 3).

**Table 3:** 30-day outcome of the cohort.

Patient characteristics	Overall	No-IMH	IMH	*p*
Mortality *n* (%)	2 (2)	1 (2)	1 (2)	1.0
New postoperative dialysis *n* (%)	1 (1)	1 (3)	0 (0)	0.4938
Stroke *n* (%)	3 (4)	2 (5)	1 (2)	0.5566
Paraplegia *n* (%)	2 (2)	1 (2)	1 (2)	1.0
ICU stay (d)	0 (0;1)	0 (0;1)	0 (0;1)	0.8659
Retrograde type A dissection *n* (%)	0 (0)	0 (0)	0 (0)	1.0
Bird beak *n* (%)	2 (2)	2 (5)	0 (0)	0.2407
Discharge with				
Beta-blockers *n* (%)	64 (79)	30 (75)	34 (83)	0.4241
ACE / AT 2 inhibitors	68 (84)	32 (80)	36 (88)	0.3793
Calcium channel blockers	63 (78)	30 (75)	33 (80)	0.6015
Endoleaks Type I *n* (%)	0 (0)	0 (0)	0 (0)	1.0

IMH, intramural hematoma; ICU, intensive care unit; ACE, angiotensin-converting enzyme; AT, angiotensin.

### Mid-term follow-up outcomes

During the follow-up of 22 (4; 43) months, 2 more patients died (2/82; 2%) of whom one patient at 3 months with prosthesis infection, while the second had lethal bleeding from an aorto-bronchial fistula at 40 months (Table 4). Strokes, paraplegia, type I/III endoleaks, or RTAD were not observed during the follow-up. Nine patients from the no-IMH group and 7 from the IMH group have undergone a reintervention which consisted of a TEVAR extension in order to repair d-SINE, which resulted in an overall reintervention rate of 19%. Bird-beak rate significantly increased in the overall cohort during the mid-term follow-up from 2/84 to 22/82 (*P* < 0.0001), without any relevant difference between the groups (No-IMH:12/41; IMH: 10/41; *P* = 0.8036). Migration of the proximal stent-graft >10 mm was seen in 21/82 patients and the groups were equal regarding this parameter (No-IMH:10/41; IMH: 11/41; *P* = 1.0).

**Table 4:** Mid-term outcome of the cohort.

Patient characteristics	Overall	No-IMH	IMH	*p*
Follow-up time (months)	22 (4;43)	20 (4; 43)	29 (4;44)	0.8799
Mortality *n* (%)	2 (2)	1 (2)	1 (2)	1.0
Aorta related mortality	2 (2)	1 (2)	1 (2)	1.0
Endoleaks type I (total) *n* (%)	0 (0)	0 (0)	0 (0)	1.0
Stroke *n* (%)	0 (0)	0 (0)	0 (0)	1.0
Paraplegia *n* (%)	0 (0)	0 (0)	0 (0)	1.0
Retrograde type A dissection *n* (%)	0 (0)	0 (0)	0 (0)	1.0
Bird beak *n* (%)	22 (27)	12 (29)	10 (24)	0.8036
Reinterventions	16 (19)	9 (22)	7 (17)	0.78132
Median bird beak angle increase during follow-up (°)	40 (34; 52)	40 (30;50)	41 (34;54)	0.6924
Migration >10 mm *n* (%)	21 (26)	10 (25)	11 (27)	1.0
Descending aortic diameter remodeling (mm)	2 (−1;5)	0 (−2;3)	3 (0;5)	0.0436
True lumen descending aortic remodeling (mm)	9 (3;15)	14 (4; 19)	7 (2;15)	0.0225
False lumen patency				
Patent	0	0	0	0
Thrombosed	80 (98)	41 (100)	39 (95)	0.1521
Partially thrombosed	2 (2)		2 (5)	0.1521

IMH, intramural hematoma.

Complete false lumen thrombosis was present in 80/82 descending thoracic aortas, with only two patients with partial thrombosis in the IMH group. The remodeling of the true lumen was more prominent in the No-IMH group with a diameter increase from preoperative to the follow-up measurement of 14 (4; 19) mm. In comparison, the diameter increase in the IMH group was 7 (2; 15) mm (*P* = 0.0225). The aortic diameter remodeling (reduction) was less pronounced in the overall cohort 2 (−1; 5) mm, with a significantly better remodeling in the IMH group (No-IMH:0 (−2;3); IMH: 3 (0;5); *P* = 0.0436).

### Risk of stent-graft migration

The multivariate logistic regression was performed to evaluate the risk of stent-graft migration, and the results are shown in Table 5. The “whole model test” was statistically significant (*P* = 0.001). The bird beak formation was the main risk factor for stent-graft migration (OR 13.3; CI 2.9–59.3.5; *P* = 0.0007; Table 5) followed by the dSINE occurrence (OR 11.1; CI 2.1–58.4; *P* = 0.0045). Stent-graft type (*P* = 0.5410), treatment zone (*P* = 0.2738), arch type (*P* = 0.7859), the timing (dissection phase) of TEVAR (*P* = 0.1333), true lumen (*P* = 0.9227) and aortic diameter (*P* = 0.9892), remodeling, and the presence of IMH in the proximal sealing zone (*P* = 0.4822) were not significant in the multivariate logistic regression. Interestingly, proximal oversizing ≤5% was not significant in the Wald test (*P* = 0.0614), however, significant in the likelihood ratio test (*P* = 0.0450), and stood out with the highest odds ratio (OR 21.5; CI 0.9–493.8).

**Table 5:** Multivariate logistic regression to evaluate the risk factors of stent-graft migration.

Patient characteristics	OR	CI	*P* (Wald test)	Likelihood ratio test
Bird beak formation	13.3	3.9–59.3	0.0007	0.0002
dSINE occurrence	11.1	2.1–58.4	0.0045	0.0023
Proximal oversizing ≤5%	20.2	0.9–471.5	0.0614	0.0450
Arch treatment zone (I + II)	3.2	0.4–25.5	0.2738	0.2882
Arch type (III)	1.2	0.3–4.7	0.7859	0.7855
Hyperacute + acute dissection	3.3	0.7–15.3	0.1333	0.1137
Relay NBS stent-graft	2.6	0.1–51.5	0.5410	0.5149
IMH in the proximal sealing zone	1.6	0.4–6.6	0.4822	0.4784
Pronounced TL remodeling (>5 mm)	0.9	0.2–4.3	0.9227	0.9228
Pronounced aortic remodeling (>5 mm)	1.01	0.2–4.7	0.9892	0.9892

OR, odds ratio; CI, confidence interval; dSINE, distal stent-induced new entry; IMH, intramural hematoma; TL, true lumen.

## Discussion

Theoretically, the anticipated proximal TEVAR landing zone in TBD may consist of a completely healthy or in extremo a totally dissected aortic wall. The latter is not regarded as an adequate and sustainable proximal landing zone even though the primary entry tear may be initially covered and the false lumen thrombosis induced. The dissected proximal landing zone may lead to proximal SINE, stent-graft migration, and type IA endoleak in the short or long-term (18). Therefore, current ESVS guidelines recommend landing in a healthy portion of the aorta ≥20 mm in length (1). However, the safety of proximal TEVAR landing in the IMH in acute/subacute TBD has rarely been reported (8). Previous reports have shown the efficacy and safety of retrograde type A IMH treatment with proximal landing in the IMH. Those smaller inhomogenous cohorts were successfully treated by TEVAR and had comparable results with open surgery going hand in hand with less postoperative morbidity (19, 20, 21). Other authors reported the occurrence of RTAD after the TEVAR treatment of the type B IMH with a diseased proximal sealing zone (affected by the IMH) (22).

The current study reports the high incidence of IMH in the proximal TEVAR landing zones in TBD, compares the outcomes of patients with and without the IMH at the proximal stent-graft end, and reports comparable results regarding RTAD, bird-beak formation, and stent-graft migration.

Our report goes in line with the previous study by Kuo et al. with considerable rates of IMH in the arch landing zones 0- III (8). The authors showed that 37% of their cohort needed zone 0 or zone I debranching to achieve at least a 10 mm IMH-free proximal TEVAR landing zone (8). Furthermore, they suspected that the occurrence of 3 RTADs may have been associated with the IMH at the proximal stent-graft end, without proving this in the multivariate analysis, due to the low patient and event numbers (8). The colleagues, therefore, recommended further evaluation of these findings in greater cohorts. We included 84 patients, and unlike Kuo et al. we excluded chronic TBD from the analysis. Furthermore, this study reports the results of a cohort with a relatively high proportion of patients treated in the hyperacute phase (27%), who may be at a higher risk of RTAD due to the fragility of the aortic wall as reported previously (4, 8). RTAD did not occur in this cohort, which may imply the safety of the proximal landing in the IMH with a moderate oversizing of approximately 10% as used in this study. Previously, oversizing of 0%–10% was recommended for the treatment of TBD to reduce the risk of RTAD (5). However, the uncertainty of the IMH fate during the follow-up (7), the result of the hematoma absorption, and its unclear effect on the dilatation of the proximal landing zone may suggest that a targeted oversizing of 10%, as applied in our study, may be reasonable in IMH-affected proximal landing zones. This is even more important due to the result of our multivariate regression analysis, which isolated an oversizing of 0%–5% as a risk factor for future stent-graft migration (OR 20.2). Furthermore, in the short term, the extent of the IMH was stable in the patients treated in the subacute phase, thus the delay of the TEVAR to the subacute phase for the purpose of IMH absorption may not be advisable. As shown by Evangelista et al. for type B IMH the absorption of the hematoma may be expected at 6 months (7) after diagnosis.

A significant increase in bird-beaks was observed in our study during the follow-up. Bird-beaks have been reported to increase the risk of type I endoleaks after TEVAR and they may lead to the instability of the stent-graft in the proximal landing zone with migration (23, 24). However, no type IA endoleaks were observed in our study and substantial migration ≥10 mm was found in 26% of the overall cohort, however, without any difference between the groups (*P* = 1.0). Furthermore, the increase of bird-beaks in the IMH and the no-IMH groups during the follow-up was equal (+10 cases). These observations may suggest that the presence of the IMH at the proximal stent-graft end does not affect the stent-graft stability in the proximal sealing zone and that migration and bird-beaks may be somewhat associated with other effects. These observations were confirmed by the multivariate analysis to identify the risk factors for migration ≥10 mm. Proximal landing in the IMH was not relevant nor were other factors including stent-graft type, treatment zone, arch type, treatment phase, and pronounced remodeling. The major risk factors for migration were the bird-beak occurrence during the follow-up (*P* = 0.0007), the occurrence of dSINE (*P* = 0.0045), and the proximal oversizing ≤5% (*P* = 0.0614).

The aortic remodeling of the true lumen was superior in the no-IMH group, which, however, may be explained by the substantially higher rate of subacute dissections with stiffer dissection membranes in the IMH group (25). Regarding the aortic diameter regression after TEVAR, the IMH group showed significantly better remodeling. Our previous works described a better diameter remodeling of subacute/chronic dissections than of those treated in the acute phase, which may be an explanation due to the higher rate of subacute dissections in the IMH group (26).

This study has several limitations. The findings of this retrospective observational study need to be confirmed by studies with a robust prospective design. Although the measurements and the study outcomes were standardized and well-defined our study may be susceptible to bias due to its retrospective and single-center design. Furthermore, the study included a limited patient number and thus, may be underpowered to determine the risk of events with a low incidence as RTAD. Nevertheless, we consider that this study may be helpful for further evaluations e.g., in a meta-analysis, due to its well-defined outcome parameters and reporting standards.

In conclusion, this study implies that the treatment of type B aortic dissections with TEVAR in the early dissection phases may be safe with a low risk of RTAD and considerable aortic remodeling in the thoracic aorta. The presence of the IMH at the proximal stent-graft end may not affect the TEVAR performance in the proximal landing zone in terms of bird-beak and migration. dSINE and bird-beak occurrence, as well as the proximal stent-graft oversizing ≤5% were identified as major risk factors for stent-graft migration.

## Data Availability

The original contributions presented in the study are included in the article/Supplementary Material, further inquiries can be directed to the corresponding author/s.
